# Introducing AI-generated cases (AI-cases) & standardized clients (AI-SCs) in communication training for veterinary students: perceptions and adoption challenges

**DOI:** 10.3389/fvets.2024.1504598

**Published:** 2025-02-24

**Authors:** Elpida Artemiou, Sarah Hooper, Linda Dascanio, Marcelo Schmidt, Guy Gilbert

**Affiliations:** ^1^Texas Tech University School of Veterinary Medicine, Amarillo, TX, United States; ^2^Department of Biomedical Sciences, Ross University School of Veterinary Medicine, St. Kitts, West Indies; ^3^Arkansas State University College of Veterinary Medicine, Jonesboro, AR, United States

**Keywords:** artificial intelligence (AI), ChatGPT, prompt engineering, communication skills, veterinary medical education, standardized clients (SCs), AI-standardized clients (AI-SCs), AI-cases

## Abstract

**Introduction:**

The integration of Artificial Intelligence (AI) into medical education and healthcare has grown steadily over these past couple of years, though its application in veterinary education and practice remains relatively underexplored. This study is among the first to introduce veterinary students to AI-generated cases (AI-cases) and AI-standardized clients (AI-SCs) for teaching and learning communication skills. The study aimed to evaluate students' beliefs and perceptions surrounding the use of AI in veterinary education, with specific focus on communication skills training.

**Methods:**

Conducted at Texas Tech University School of Veterinary Medicine (TTU SVM) during the Spring 2024 semester, the study included pre-clinical veterinary students (*n* = 237), who participated in a 90-min communication skills laboratory activity. Each class was introduced to two AI-cases and two AI-SCs, developed using OpenAI's ChatGPT-3.5. The Calgary Cambridge Guide (CCG) served as the framework for practicing communication skills.

**Results:**

Results showed that although students recognized the widespread use of AI in everyday life, their familiarity, comfort and application of AI in veterinary education were limited. Notably, upper-year students were more hesitant to adopt AI-based tools, particularly in communication skills training.

**Discussion:**

The findings suggest that veterinary institutions should prioritize AI-literacy and further explore how AI can enhance and complement communication training, veterinary education and practice.

## 1 Introduction

The study of mathematically explaining in detail the process of human learning and intelligence such that a machine can replicate was first described in 1955 by John McCarthy (1). Around this time, the term Artificial Intelligence (AI) was coined to describe the scientific method of developing computer algorithms that simulate human cognition (2, 3). The integration of AI has progressed more rapidly in human healthcare compared to veterinary medicine (4, 5). Presently in human medicine, AI-based solutions support clinical-decision making (6, 7), facilitate the understanding and analysis of written and verbal human language and generate appropriate dialogue as experienced in Chatbots (8), have the capacity process unstructured clinical text to generate predictive clinical outputs (9) and offer diagnostic tools to prevent disease (10). In veterinary medicine, AI has been used successfully in the areas of radiology (4, 11), disease surveillance, diagnosis and decision-making process (12, 13).

Despite significant advances and changes brought about by AI across various healthcare professions, veterinary medicine professionals remain skeptical about its applicability and the necessity to equip students with AI-related knowledge and skills (14, 15). Only a few examples in veterinary medical education have embraced AI, such as using it to support student learning, teach veterinary anatomy, and assess clinical skills (5, 16, 17).

Emerging technologies, evolving clients' and societal expectations are pushing for a paradigm shift in veterinary medical education. The growing emphasis on competency-based education, grounded in the principles of andragogy and student-centered learning (18) highlights the need to prepare veterinary graduates for a rapidly changing professional landscape. In response veterinary graduates from member institutions of the Association of American Veterinary Medical Colleges (AAVMC) are expected to meet minimum competencies across nine domains including clinical reasoning and decision-making, individual animal care and management, animal population care and management, public health, communication, collaboration, professionalism and professional identity, financial and practice management and scholarship (19, 20).

To ensure that students meet these competencies, veterinary education has increasingly integrated experiential learning methodologies. Kolb's experiential learning theory, for example, has proven effective in enhancing students' skills and confidence in clinical communication (21). By incorporating techniques such as role-play, practice with Standardized Clients (SCs), structured and constructive feedback, and presentation of skills in a helical approach along with repeated practice; all approaches align to develop well-rounded, competent veterinary professionals (22–25). Among the different approaches to communication skills training, the Calgary Cambridge Guide is a validated framework for teaching and assessing communication skills in veterinary education and has been widely used with a focus on relationship-centered medicine and effective and compassionate client interactions (26, 27).

Communication curricula are resource intensive requiring administrative support, recruitment and training of SCs and facilitators, continuing case development and refinement, investment in audiovisual software and facilities (23, 28). Limited studies across healthcare professions report the use of AI in communication skills training; AI-examples include virtual patients and Chatbots to practice the clinical interview and to provide feedback (29, 30) yet additional use of AI can potentially complement communication teaching, learning and assessment while reducing costs and increasing efficiency.

This is the first study to explore the integration of AI in a veterinary communication pre-clinical Doctor of Veterinary Medicine (DVM) program (5, 13). The study aimed to introduce veterinary students to AI-developed cases and AI-simulated clients (AI-SCs) and examine students' perceptions regarding the use of AI in veterinary education, particularly within communication skills training programs.

## 2 Materials and methods

### 2.1 TTU SVM curriculum

The Texas Tech University School of Veterinary Medicine (TTU SVM) follows an outcome and competency based 3-year pre-clinical curriculum followed by a 1-year practice through a community based clinical learning network around Texas and parts of New Mexico. The clinical and professional skills program (CPS) engages students in 6 hours weekly of hands-on experiential practice across small animal, food animal, equine and exotic species and organized across three focus areas: surgery, medicine and communication skills. Specifically, communication skills are delivered in three 50-min active, student-centered presentations where students are presented theoretical frameworks and evidence-based delineation of communication skills followed by three 3-h communication laboratories per semester across all three pre-clinical years. Communication teaching, learning outcomes and assessment is based on the Calgary Cambridge Guide (CCG) (25, 26). The CCG delineates 73 process skills and outlines the clinical interview into five sequential stages: initiating the session, gathering of information, physical examination, explanation and planning, closing the session, and two stages that run throughout the encounter: building rapport and providing structure. In line with the Calgary-Cambridge Guide (CCG), students participate in laboratory sessions where they work in groups of 4–5, alongside a facilitator, to practice communication skills in simulated encounters with standardized clients (SCs). During these sessions, students receive real-time feedback while engaging in self-assessment and reflective practices to enhance their communication skills.

With advancements in AI rapidly transforming various fields, its application in veterinary education, particularly in communication skills training, remains largely unexplored. This study, conducted during the Spring semester of 2024, represents a pioneering effort to integrate AI into the development of veterinary communication skills. All students (*n* = 243) enrolled in semesters two (*n* = 102), four (*n* = 79), and six (*n* = 62) were invited to practice communication skills with AI-generated cases and AI-standardized clients (AI-SCs) as part of a course assignment. By examining students' perceptions regarding the use of AI in veterinary education, this research offers valuable insights into the potential of AI to enhance communication training.

The study was approved by the Institutional Review Board (IRB) at Texas Tech University, IRB # 2023-1125.

### 2.2 AI-cases

We developed six AI-cases using the free conversational OpenAI Chat Generative Pre-Trained Transformer version, ChatGPT-3.5. ChatGPT is trained to retrieve information from large datasets which enables adding depth and contextual nuances to clinical scenarios that can be used in experiential practice of communication skills (31). The AI cases were incorporated into semesters 2, 4, and 6 during the 2^nd^, 3^rd^, and 14^th^ weeks of the respective semesters to align with and support the learning objectives of the labs and communication curriculum. The cases developed were designed to engage learners on addressing animal health communication and how best to address human differences including disability, illness, exposure to various racial and ethnic backgrounds, sexual orientations, socioeconomic status, and other demographic differences that students may encounter when they enter veterinary medical practice. All human characteristics were presented to the learner as part of the patient and client history before the exercise and interaction.

### 2.3 Prompt engineering

A prompt is a natural language text that describes a task that an AI should perform in response (32) while prompt engineering describes the process of structuring an instruction that can be interpreted and understood by AI (33). For our study, the following prompt engineering technique was utilized to create and enrich cases with desired social and cultural characteristics; Name of the Owner: [INSERT NAME] Ethnicity: [INSERT ETHNIC GROUP] Location: [INSERT DEPARTMENT, RURAL/URBAN AREA] Language Spoken: [INSERT LANGUAGE] Occupation: [INSERT OCCUPATION] Household Composition: [INSERT HOUSEHOLD COMPOSITION] Type of Animal: [INSERT ANIMAL TYPE] Animal's Name: [INSERT ANIMAL NAME] Animal's Age: [INSERT AGE] Reason for Veterinary Visit: [INSERT REASON] Possible Socioeconomic or Cultural Factors Affecting the Case: [INSERT FACTORS]. Next, the scenarios were further enhanced with images utilizing photo animating application Talkr Inc. (2023) Talkr Live version 2.3 and voice over changing hardware Roland Corporation (2018) VT-4 voice transformer.

### 2.4 AI-intervention

We designed a 90-min communication laboratory to practice communication skills following an individualized and reflective approach. The AI generated cases were hosted on Blackboard Inc. Learning Management System (LMS) accessed through the TTU SVM intranet. The laboratory encompassed a 30-min theory and evidence followed by 60 min of practice of communication skills. The theoretical component provided information on the evidence and theory of communication skills and the use of AI in teaching and learning communication and a review of core communication skills. The practical component was based on the premises of the CCG; students watched and listened the AI-Standardized Client (SC) case and were asked to develop a dialogue for a clinical veterinary encounter following the CCG framework with specific attention to the AI-SC narrative. The practice session included several pauses, allowing students to ask questions and enabling two facilitators to provide clarifications as needed. Students were encouraged to work in pairs and practice their developed dialogue; one student took on the role of the “writer” and wrote out specific communication skills while the other student focused on being the “listener” and provided feedback on how skills were practiced. Both the “writer” and the “listener” contributed to developing the dialogue. Students were encouraged to write out in words how non-verbal communication skills would be practiced and applied during the encounter.

### 2.5 Semester 2 AI-SCs

The AI cases for semester 2 students focused on developing skills aligned with the Calgary-Cambridge Guide (CCG), specifically targeting the stages of initiating the consultation, gathering of information, building rapport, and providing structure. Additionally, students had the opportunity to center communication around socioeconomic and cultural factors.

**Figure 1:**
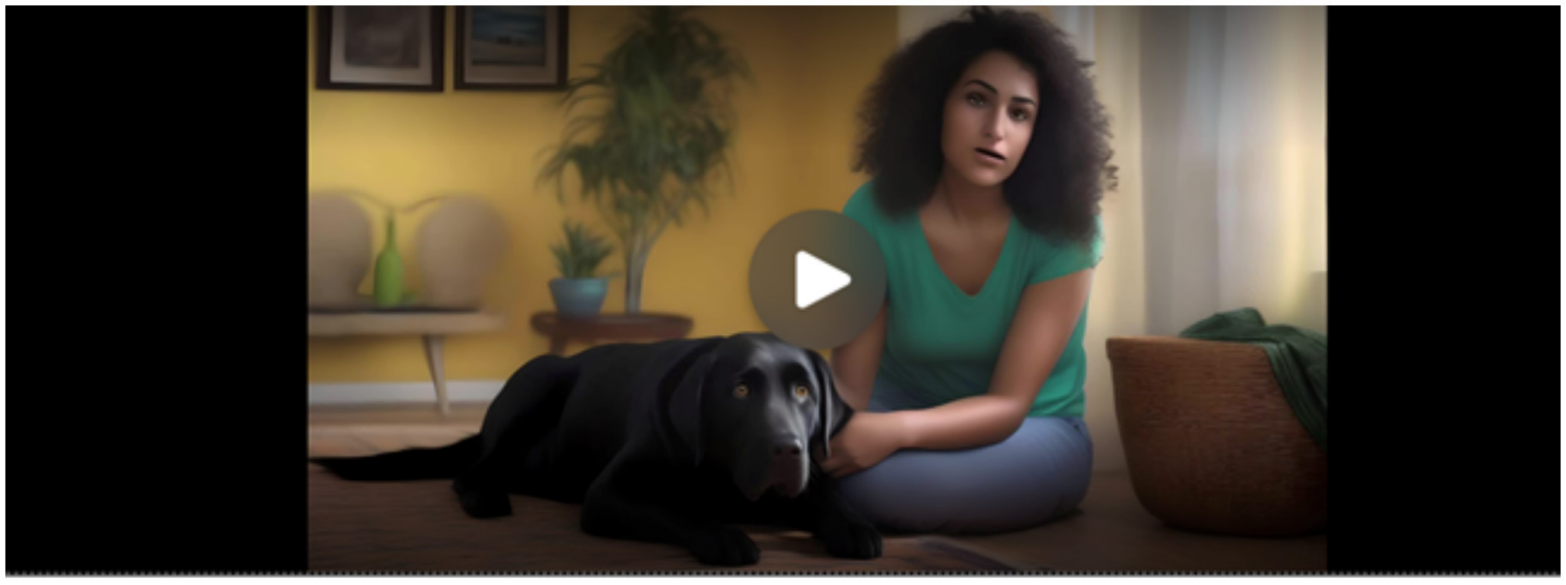
Still image of AI-SC Suzanne Ouais, a student of American/Lebanese descent, with her 14-year-old Black Lab mix with diarrhea.

AI-SC Suzanne Ouais (Figure 1) presented with her 14-year-old Black Lab mix with diarrhea. Suzanne Ouais is a student of American/Lebanese descent who recently relocated to Lubbock, from the Caribbean with her beloved 14-year-old mixed Black Lab, Pippa. Suzanne's limited finances and reliance on student loans make her budget-conscious and she struggles to meet all Pippa's healthcare needs. Suzanne is worried about Pippa's health especially as she is getting older and wants to ensure that she can provide the necessary care within her budget.

**Figure 2:**
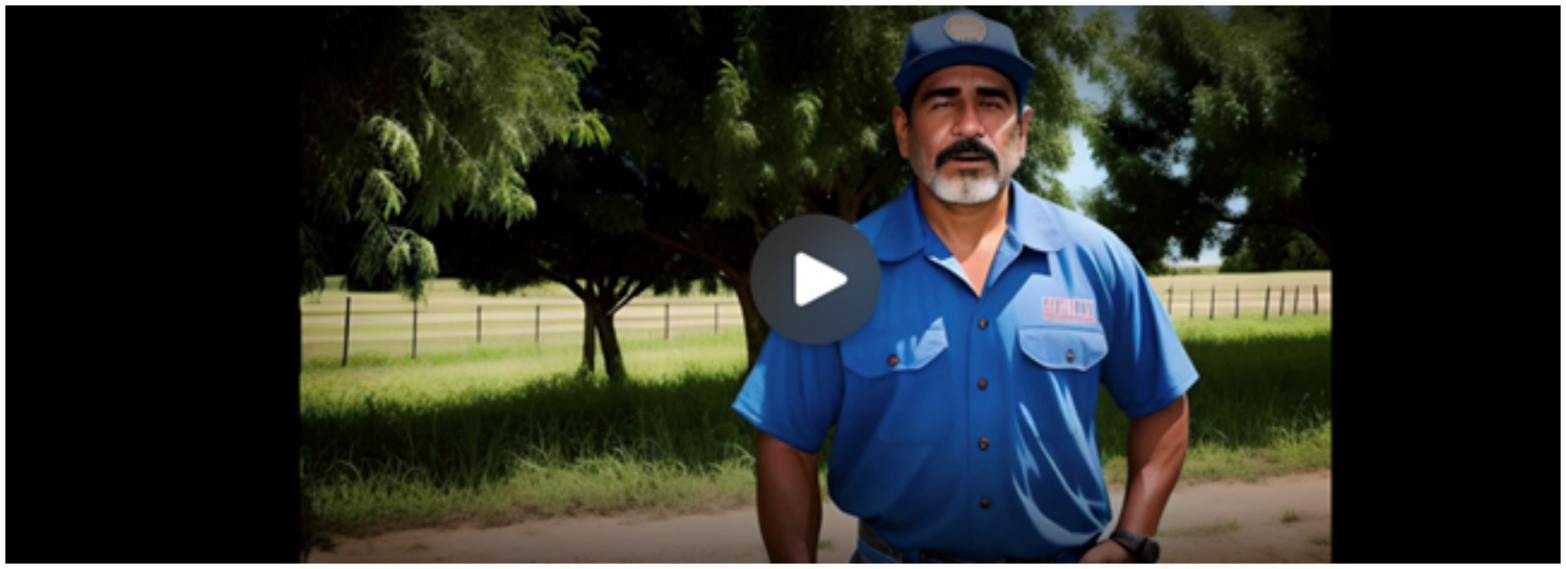
Still image of AI-SC Erik Garcia, a dairy owner of Latino descent, seeking a second opinion for purchasing a 200-head dairy farm.

AI-SC Erik Garcia (Figure 2) presented for a second opinion for purchasing a 200-head dairy farm. Students were encouraged to consider effective and compassionate communication to address socioeconomic, cultural factors and language barriers.

Erik Garcia is a hardworking and family-oriented individual of Latino background and resides in the rural outskirts of Amarillo, Texas, where he and his family have been living for several years. Erik shared his strong commitment to ensuring the health and welfare of the dairy herd. He wants to provide top-quality care for his animals and ensure the success of the farm to support his family. Erik is bilingual, speaking both Spanish and English fluently, but he is more comfortable expressing himself in Spanish.

### 2.6 Semester 4 AI-cases

The AI cases for semester 4 students focused on developing skills aligned with the Calgary-Cambridge Guide (CCG), specifically targeting the stages of initiating the consultation, explanation, and planning, building rapport, and providing structure. Students were encouraged to explore the client's concerns and perspective.

**Figure 3:**
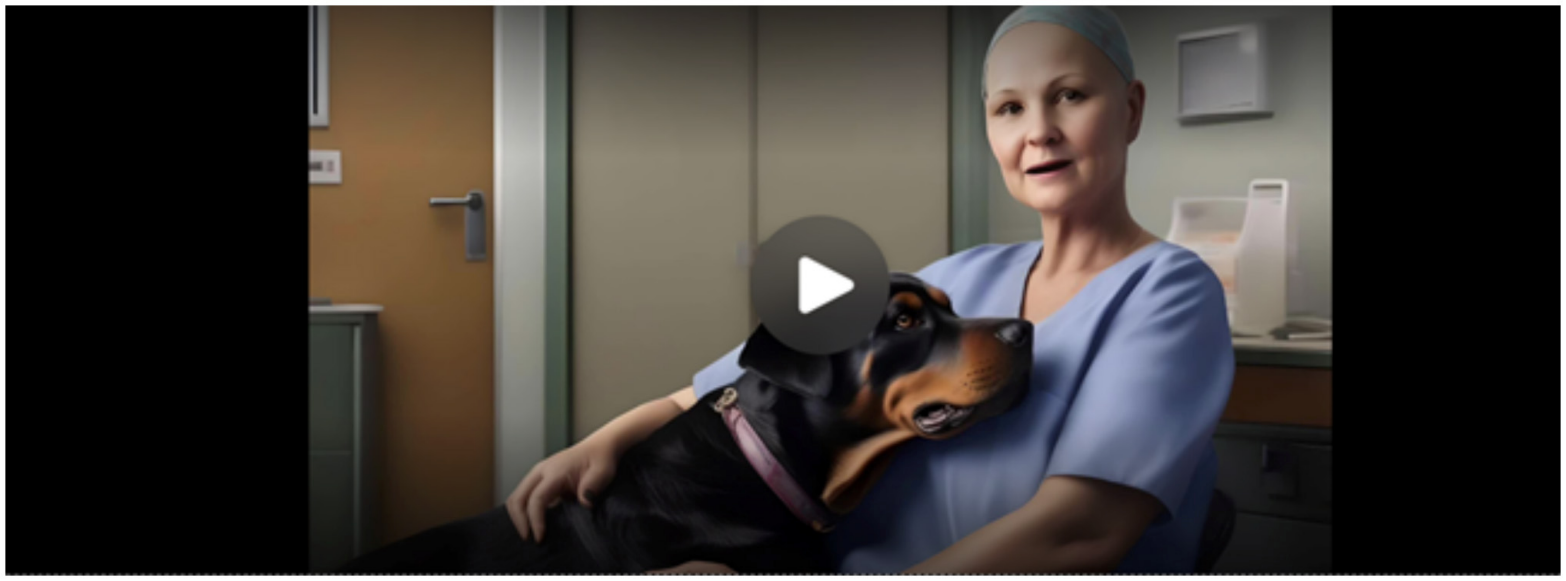
Still image of AI-SC Rebekka Stone, a mother and high-school math teacher with stage 4 breast cancer, with her 6-year-old Rottweiler recently diagnosed with osteosarcoma.

AI-SC Rebekka Stone (Figure 3) presented with her 6-year-old neutered Rottweiler, Rambo, to receive test results for a diagnosis of osteosarcoma. Rebekka Stone, a mom to three children, a high-school math teacher is facing multiple challenges in her life. She has been diagnosed with stage 4 breast cancer and is currently undergoing chemotherapy. These factors impact her ability to provide the best care for her beloved Rottweiler, Rambo, who has been diagnosed with osteosarcoma. The costs associated with diagnosing and treating Rambo's cancer is a major concern for Rebekka, given her own ongoing medical expenses. Furthermore, Rebekka's chemotherapy treatments may limit her availability, ability and energy to care for Rambo during this challenging time.

**Figure 4:**
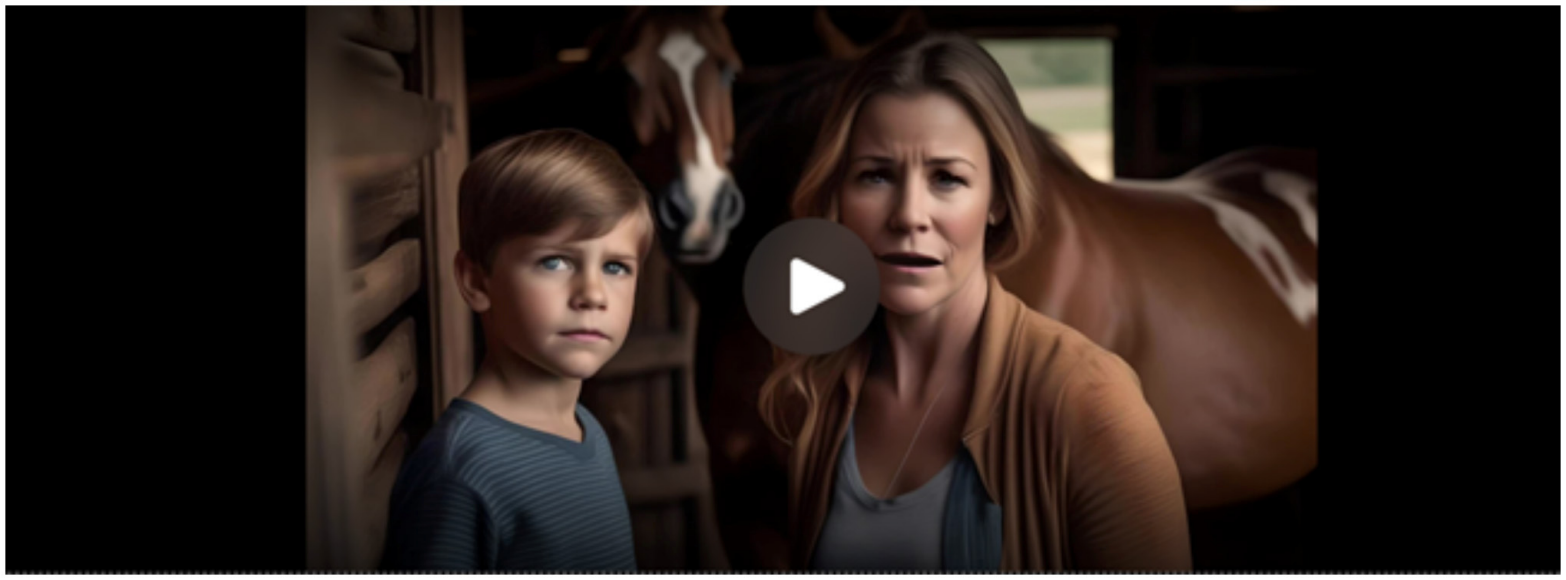
Still image of AI-SCs 8-year-old Peter, an autistic child, and his mom Anne Black with Daisy Bell, a therapy horse, presenting for signs of colic.

AI-SCs 8-year-old Peter and his mom Anne Black (Figure 4) presented with DaisyBell, a therapy horse, for signs of colic. Students were encouraged to discuss the potential diagnosis of colic while exploring how best to accommodate and meet Peter's needs. Peter is diagnosed with autism and has difficulty expressing his feelings and struggles to understand what is happening with DaisyBell. Students were encouraged to use visual aids in discussing the diagnosis, treatment and risks.

### 2.7 Semester 6 AI-cases

The AI cases for semester 6 students focused on developing skills aligned with six stages of the Calgary-Cambridge Guide (CCG), initiating the consultation, gathering of information, explanation, and planning, building rapport, providing structure, and closing the consultation. Students were encouraged to explore communication skills that support compassionate care when working with vulnerable populations.

**Figure 5:**
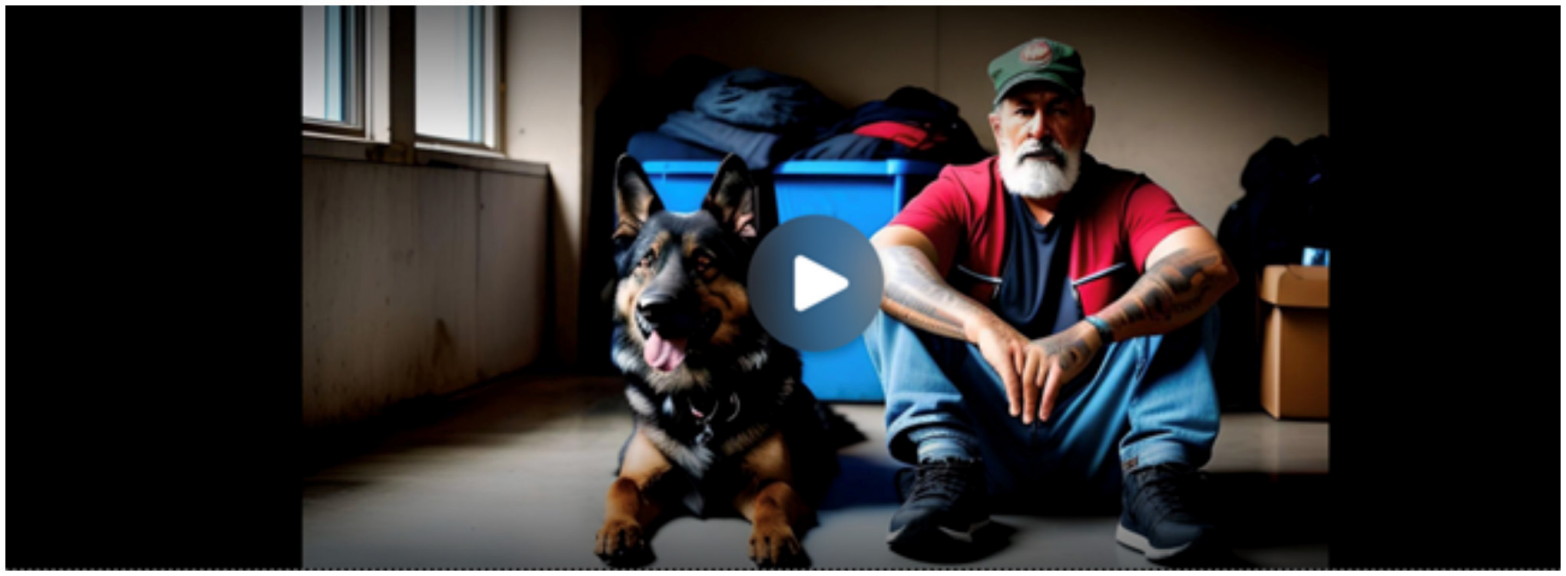
Still image of AI-SC Danny Erickson, a homeless marine veteran diagnosed with Post-Traumatic-Stress-Disorder (PTSD), with his 10-year-old dog Rocket presenting for hind limb lameness and potential hip dysplasia.

AI-SC Danny Erickson (Figure 5) and his 10-year-old dog, Rocket, was seen for hind limb lameness and a potential diagnosis of hip dysplasia. Danny Erickson is a marine veteran diagnosed with Post-Traumatic-Stress-Disorder (PTSD) and now homeless. Danny has a history of being in and out of the veteran's hospital, also impacting his ability to consistently care for Rocket, as he may have periods of hospitalization or treatment that prevent him from providing adequate care.

**Figure 6:**
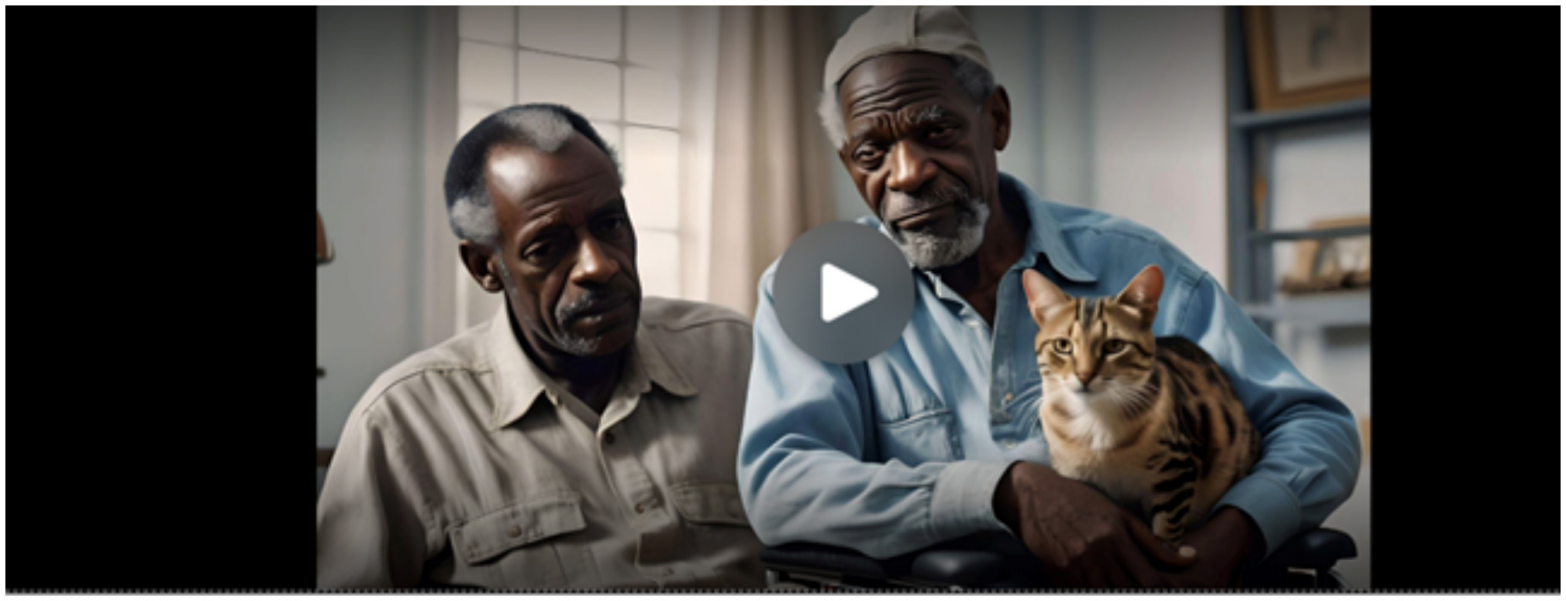
Still image of AI-SCs Phillip Nichols, a client diagnosed with HIV, and his partner Marcus Stephen, with their 13-year-old Savannah cat Chloe presenting with hypertrophic cardiomyopathy.

AI-SCs Phillip Nichols and his partner Marcus Stephen (Figure 6) are seen with their 13-year-old Savannah cat, Chloe, that is diagnosed with hypertrophic cardiomyopathy. Students were encouraged to discuss medical care for Chloe while considering the client's unique circumstances and medical condition. Phillip receives palliative care for Human Immunodeficiency Virus (HIV) and is very frail with limited mobility, which impacts on his ability to provide comprehensive care for Chloe.

### 2.8 Questionnaire

We utilized enterprise survey software to create and distribute an online questionnaire to understand veterinary students' perceptions regarding the use of AI and technology in their veterinary education and communication training. The questionnaire was piloted with veterinary and graduate students, and veterinary educators who were independent from the course. The final version of the survey included (i) 4 broad questions that established the student's semester of study; area of interest in veterinary medicine; age and gender, and (ii) four sections with 5-point Likert scale responses. The first section included nine questions that explored technology literacy and use in veterinary education (e.g., How often do you find yourself turning to software or apps to help with your coursework?, “Almost never = 0” to “All the time = 5”) (Table 1). Section 2 covered seven questions that investigated the student's experience with AI and GPTs (e.g., How familiar are you with the term Artificial Intelligence (AI)?, “Not at all familiar = 0” to “Very familiar = 5”) (Table 2). Section 3 comprised twelve questions that addressed students' experience with SCs and experiential practice, (e.g., How effective are encounters with Standardized Clients (SCs) as part of your veterinary training in communication skills?, “Not effective = 0” to “Very effective = 5”) (Table 3). Lastly the fourth section included 9 questions that focused on students' expectations and concerns about AI integration in veterinary education and communication (e.g., How do you think AI might change the way you learn in veterinary school?, “Not at all = 0” to “A great extend = 5”) (Table 4). Veterinary students completed the AI questionnaire at the end of the AI case experience.

**Table 1:** Pre-survey questions along with the technology and education survey section.

**Question**	**Response options**
Please specify your current semester in veterinary school.	Second
	Fourth
	Sixth
Please select your primary area of interest in veterinary medicine:	Academic and industry
	Community practice
	Equine health
	One health (public, global health & regulatory veterinary medicine)
	Production animal health
Please select your year of birth.	Free text response
Please select your gender.	Female
	Male
	Prefer not to say
**Section 1: Technology and education**	
Question 1: How much do you rely on online resources for your veterinary education?	Almost never
	Seldom
	Sometimes
	Often
	All the time
Question 2: How often do you find yourself turning to software or apps to help with your coursework?	Almost never
	Seldom
	Sometimes
	Often
	All the time
Question 3: When it comes to learning new tech tools for your classes, how confident do you feel?	Not at all
	A little
	Moderately
	Quite a bit
	A great deal
Question 4: How much do you enjoy using technology as part of your learning process?	Not at all
	A little
	Moderately
	Quite a bit
	A great deal
Question 5: How often do you independently explore new tech tools or apps beyond what is provided for your courses?	Almost never
	Rarely
	Sometimes
	Often
	All the time
Question 6: How easy is it for you to fix small issues with the digital tools you use?	Very difficult
	Difficult
	Moderate
	Easy
	Very easy
Question 7: How much have digital tools improved your learning in veterinary school?	Not at all
	A little
	Moderately
	Quite a bit
	A great deal
Question 8: How often do you feel overwhelmed by the technology skills required in your studies?	Almost never
	Rarely
	Sometimes
	Often
	All the time
Question 9: How likely are you to recommend the use of digital learning tools to your peers?	Not very likely
	Slightly likely
	Moderately likely
	Likely
	Very likely

The responses are on a 5-point Likert scale.

**Table 2:** Experience with AI and GPTs survey section.

**Section 2: Experience with AI and GPTs**	
Question 10: How familiar are you with the term “Artificial Intelligence” (AI)?	Not at all familiar
	Slightly familiar
	Moderately familiar
	Familiar
	Very familiar
Question 11: How often do you use or interact with AI tools, in any context?	Almost never
	Rarely
	Sometimes
	Often
	All the time
Question 12: How often do you think AI is involved in the technology you use every day, like social media or online shopping?	Almost never
	Rarely
	Sometimes
	Often
	All the time
Question 13: Have you noticed features like auto-correct or predictive text on your phone?	Never noticed
	Rarely noticed
	Sometimes noticed
	Often noticed
	Very noticed
Question 14: How likely are you to explore AI tools on your own for personal or educational purposes?	Not very likely
	Slightly likely
	Moderately likely
	Likely
	Very likely
Question 15: If you've used AI-driven tools, how easy was it for you to understand and use them?	Not at all easy
	Slightly easy
	Moderately easy
	Easy
	Very easy
Question 16: Rate your interest in learning more about AI and its applications in veterinary medicine.	Not at all interested
	Slightly interested
	Moderately interested
	Interested
	Very interested

The responses are on a 5-point Likert scale.

**Table 3:** Standardized client survey section.

**Section 3: Standardized clients**	
Question 17: How effective are encounters with standardized clients (SC) as part of your veterinary training in communication skills?	Not effective
	Slightly effective
	Moderately effective
	Effective
	Very effective
Question 18: What is your level of engagement during the SC encounters?	Not at all engaged
	Somewhat engaged
	Moderately engaged
	Quite engaged
	Very engaged
Question 19: How realistic do you find the SC encounters?	Not at all realistic
	A little realistic
	Moderately realistic
	Quite a bit realistic
	Very realistic
Question 20: How well do the SCs reflect the diversity of clients/owners that you might encounter in practice?	Not at all
	A little
	Moderately
	Quite a bit
	Very well
Question 21: How well do the communication scenarios reflect the diversity of cases you might encounter in practice?	Not at all
	A little
	Moderately
	Quite a bit
	A great deal
Question 22: How effective are encounters with SCs in preparing you for real-world veterinary practice.	Not effective
	Slightly effective
	Moderately effective
	Effective
	Very effective
Question 23: After participating in SC encounters, how confident do you feel in handling similar situations in real life?	Not at all confident
	Slightly confident
	Moderately confident
	Confident
	Very confident
Question 24: How likely are you to recommend the use of technologically advanced simulations in communication training?	Not at all likely
	Slightly likely
	Moderately likely
	Likely
	Very likely
Question 25: Do you think technology can enhance the SC encounters?	Not at all
	A little
	Moderately
	Quite a bit
	Very much
Question 26: Would you be interested in more technology-enhanced simulations, like virtual reality, in your communication training?	Not at all
	Slightly
	Moderately
	Interested
	Very interested
Question: 27 How beneficial do you find the feedback from instructors during the SC encounters?	Not beneficial
	A little beneficial
	Moderately beneficial
	Beneficial
	Very beneficial
Question 28: How beneficial would you consider feedback from AI during the SC encounter?	Not beneficial
	A little beneficial
	Moderately beneficial
	Beneficial
	Very beneficial

The responses are on a 5-point Likert scale.

**Table 4:** Expectations and concerns about AI integration in veterinary education and communication survey section.

**Section 4: Expectations and concerns about AI integration in veterinary education and communication**	
Question 29: Do you think AI can help personalize your veterinary learning experience?	Not at all
	A small extent
	A moderate extent
	A large extent
	A great extent
Question 30: What is your level of comfort with the idea of AI-assisted learning.	Not at all comfortable
	A little comfortable
	Moderately comfortable
	Quite comfortable
	Very comfortable
Question 31: How do you think AI might change the way you learn in veterinary school?	Not at all
	Slightly
	Moderately
	Significantly
	Very significantly
Question 32: How willing are you to experiment with AI tools in your learning process?	Not at all willing
	Slightly willing
	Moderately willing
	Willing
	Very willing
Question 33: Do you think AI could help improve your communication skills across veterinary contexts/disciplines?	Not at all
	A little
	Moderately
	Quite a bit
	A great deal
Question 34: How do you feel about the possibility of AI providing feedback on the use of your communication skills?	Not very comfortable
	Slightly comfortable
	Moderately comfortable
	Comfortable
	Very comfortable
Question 35: Do you believe AI will play an important role in the future of veterinary education?	Not at all
	Slightly
	Moderately
	Mostly
	Definitely
Question 36: Are you concerned about the privacy and/or security of your data when using AI tools?	Not at all
	A little
	Moderately
	Quite a bit
	A great deal

The responses are a 5-point Likert scale.

### 2.9 Statistical analysis

Descriptive and inferential statistics were utilized to analyze survey results. Data was collected from a student assignment. All data was stored on TTU-approved University systems requiring two-factor authentication, accessible only to IRB-approved investigators. To further protect data privacy, the analysis was conducted using a dataset with all names removed and ages assigned to generations to ensure anonymity. We assigned students born between 1965 and 1980 to “Gen X,” those born between 1981 and 1996 to “Millennials,” and those born between 1997 and 2012 to “Gen Z.”

We performed all analyses using RStudio “Chocolate Cosmos” release for macOS with R (version 4.4.0) (34). The data exhibited a non-normal distribution leading to selection of Spearman's correlation coefficient to assess correlations among all survey questions, as Winter et al. suggested for non-normally distributed data with outliers (35). We used the R packages Rstatix (version 0.7.2) and GGally (version 2.2.1) to calculate Spearman's correlation coefficient, and subsequently the corrplot package (version 0.92) to construct visualizations of the results. We followed guidelines provided by Akoglu (36) to interpret correlation coefficients as weak with a ρ = 0.10–0.39, moderate ρ = 0.40–0.69, and strong ρ = 0.70–1.

We completed Kruskal-Wallis tests, with Holm adjusted *p*-values, using the R package ggstatsplot (version 0.12.4) to determine if response differences existed between semesters, generations, and career interests. Any significant Kruskal-Wallis test was followed by Dunn's test of multiple comparisons using dunn.test package (version 1.3.6) to determine which semesters were significantly different from each other. We constructed visualizations of the Likert data using the Likert (version 1.3.5) R package.

## 3 Results

### 3.1 Demographics

Table 5 shows the age range and career interest for the 237 students who agreed to participate in the study and completed the survey. One-hundred and thirty-eight students were identified as Gen Z, 51 as Millennials, and 2 as GenX. One student did not report his/her age.

**Table 5:** Number of complete survey responses, participation rate, age range, and career interest by semester.

**Semester**	**Number of responses**	**Participation rate**	**Age range**	**Career interest**
Second	102	100%	22–47	Academic and Industry 1
				Community Practice 55
				Equine Health 30
				One Health (Public, Global Health & Regulatory Veterinary Medicine) 2
				Production Animal Health 14
Fourth	71	90%	23–39	Community Practice 39
				Equine Health 17
				One Health (Public, Global Health & Regulatory Veterinary Medicine) 1
				Production Animal Health 14
Sixth	61	98%	24–46	Community Practice 48
				Equine Health 9
				Production Animal Health 4

### 3.2 Questionnaire

#### 3.2.1 Descriptive statistics and correlations

##### 3.2.1.1 Section 1: technology and education

**Figure 7:**
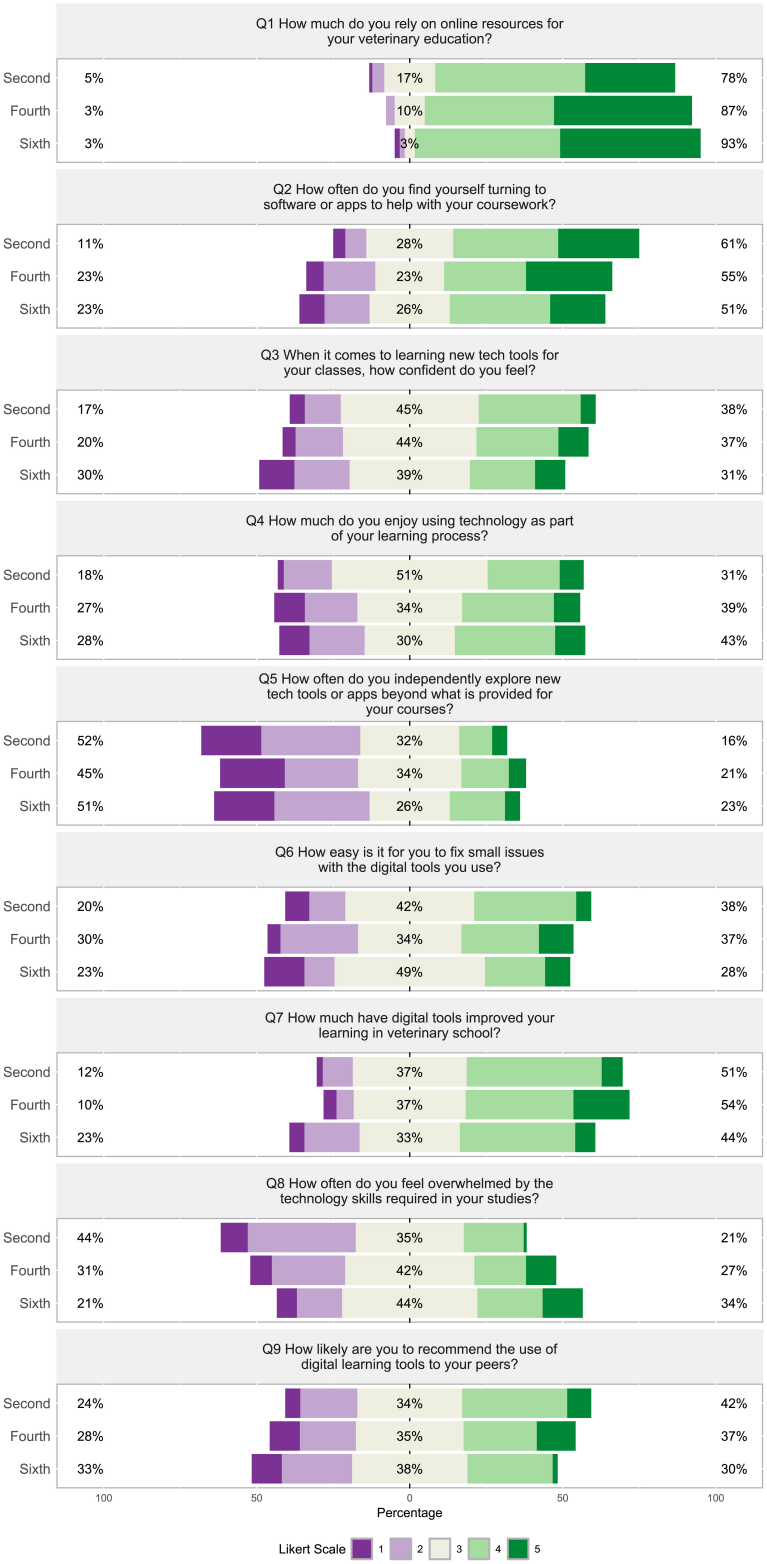
(Q1–Q9): Visualization of the distribution of student responses across different semesters using the Likert scale for responding to in Section 1: technology and education survey section. Percentages on the left are more negative responses (Likert scale value 1 and 2: e.g., “almost never” and “very difficult”). The middle section shows the percentage of neutral responses (Likert scale value 3). Percentage on the right represent the more positive responses (Likert scale value 4 and 5: e.g., “all the time” and “a great deal”). Table 1 displays the Likert response options.

Throughout all the semesters, students reported “often” or “all the time” relying heavily on online resources (78%−93%) while less often relying upon software, apps, and new tech tools for their coursework (Figure 7, Q2). Forty-five to 52% responded that they were “not rarely” to “never” independently explore new tech tools or apps beyond what is provided in the course (Figure 7, Q5). However, 33%−37% of students felt digital tools moderately improved their learning with 44%−55% reporting that digital tools improved their learning by “quite a bit” to a “great deal” (Figure 7, Q7). Forty-four percent of second semester students were “almost never” to “rarely” overwhelmed by technology skills required, compared to fourth and sixth semester students who reported 31% to 21%, respectively (Figure 7, Q8).

**Figure 8:**
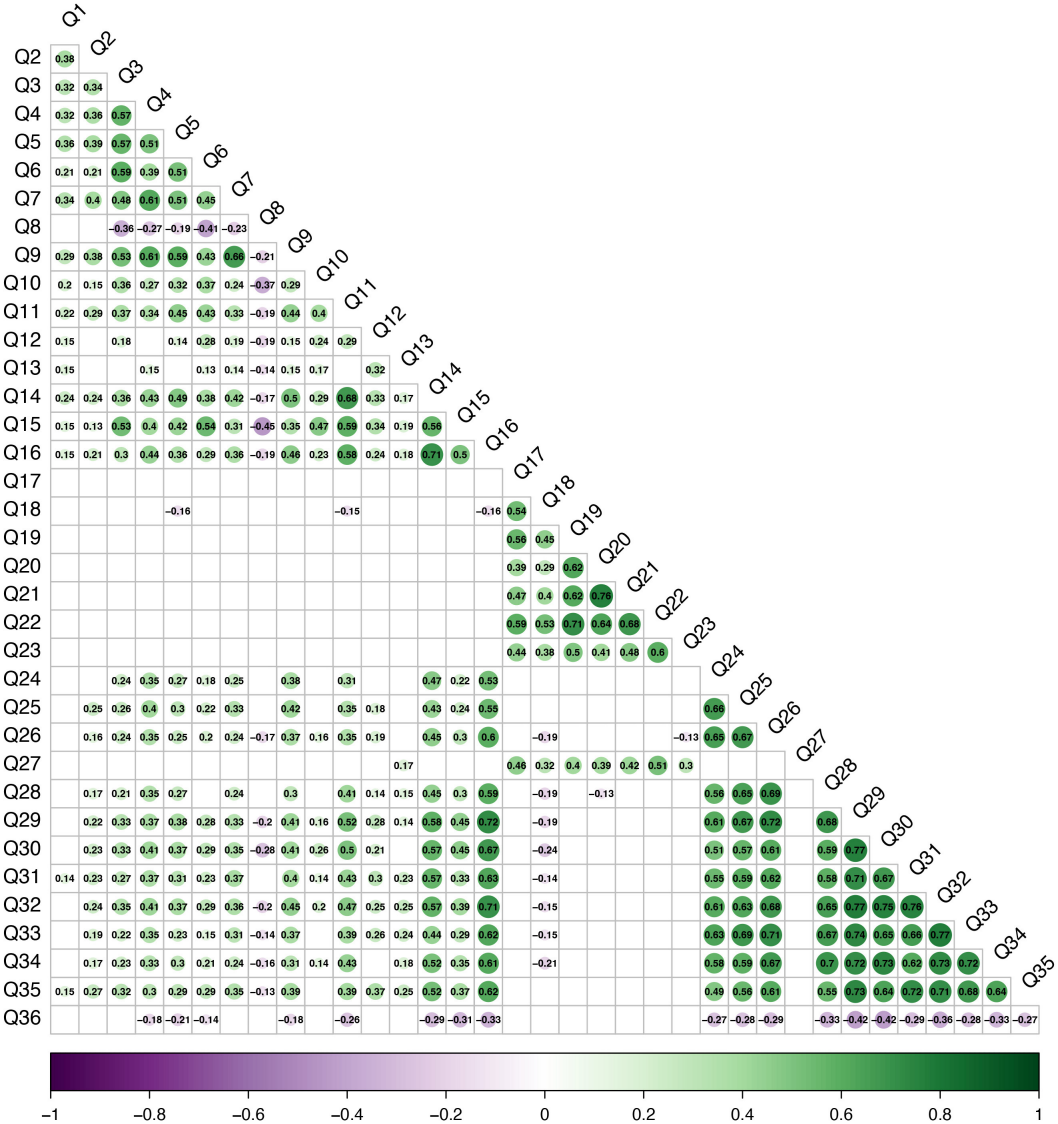
Statistically significant (*p* < 0.05) Spearman's correlation coefficients. The circle size and color scale indicate the strength of the correlation coefficient with purple representing negative coefficients and green indicating positive correlation coefficients.

Questions 1 through 7 were weakly (ρ = 0.21) to moderately (ρ = 0.61) positively correlated (Figure 8, Q1–Q7). Question 8, which focused on students feeling overwhelmed by the technology skills was weakly (ρ =−0.19) to moderately (ρ = −0.41) negatively correlated with questions 3 through 7 (Figure 8, Q8 and Q3–Q7). Weak (ρ = 0.29) to moderate (ρ = 0.66) positive correlations were appreciated between question 9, focusing on the use of digital learning tools, and questions 1 through 7 (Figure 8, Q9 and Q1–Q7). Figure 8 shows that question 8, “How often do you feel overwhelmed by the technology skills required in your studies?” was weakly (ρ = −0.14) to moderately (ρ = −0.45) negatively correlated with questions in all other sections of the survey. Question 5, “How often do you independently explore new tech tools or apps beyond what is provided for your course?” was weakly negatively correlated to the student's level of engagement during simulated client encounters (Figure 8, Q5 and Q18, ρ = −0.16) and with the student's concern about their privacy/security of their data (Figure 8, Q5 and Q36, ρ = −0.21). Figure 8 displays all significant correlations (*p* < 0.05) for all questions in the survey.

##### 3.2.1.2 Section 2: experience with AI and GPTs

**Figure 9:**
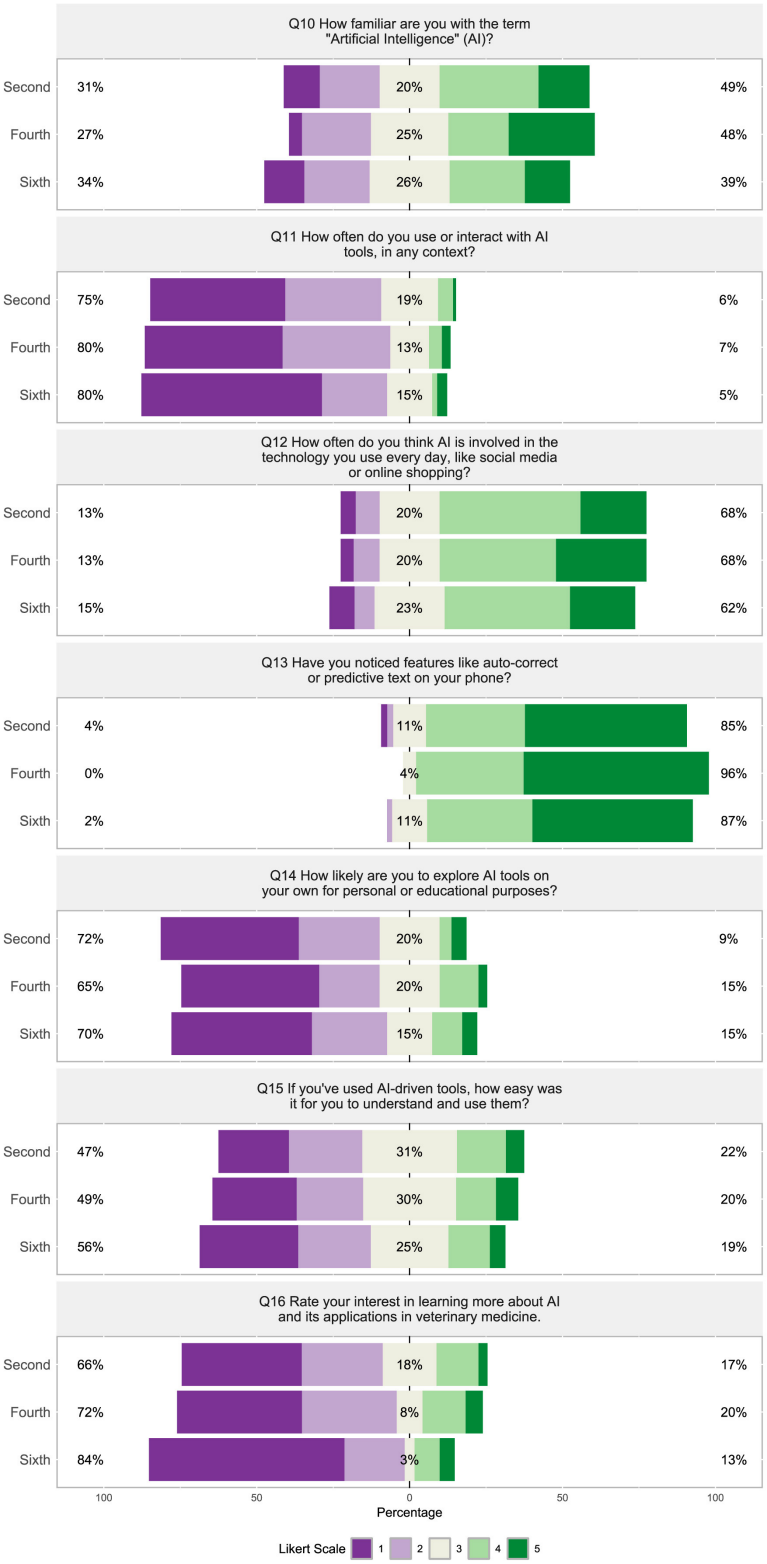
(Q10–Q16): Visualization of the distribution of student responses across different semesters using the Likert scale for responding to questions in the technology in Section 2: experience with AI and GPT survey section. Percentages on the left are more negative responses (Likert scale value 1 and 2: e.g., “almost never” and “not at all easy”). The middle section shows the percentage of neutral responses (Likert scale value 3). Percentage on the right represent the more positive responses (Likert scale value 4 and 5: e.g. “very familiar” and “very likely”). Table 2 displays the Likert response options.

Students across all semesters ranged from “not at all familiar” to “very familiar” with the term “Artificial Intelligence” (AI) (Figure 9, Q10). The majority of students (75%−80%) “almost never” or “rarely” use or interact with AI tools in any context (Figure 9, Q11) and 65%−72% were “not very likely” or only “slightly likely” to explore AI tools on their own for personal or educational purposes (Figure 9, Q14). When students used AI-driven tools, around half (47%−56%) found them “not at all easy” or “slightly easy” to understand and utilize them (Figure 9, Q15), and most students (66%−84%) were “not at all interested” or “slightly interested” in learning more about AI and its applications in veterinary medicine (Figure 9, Q16). Sixty-two to 68% of students reported that AI is “often” to “all the time” involved in everyday technology (Figure 9, Q12) and the majority of students (85%−96%) “often” or “very often noticed” features like predictive text on their phones (Figure 9, Q13).

Students' opinions about exploring AI tools strongly correlated to their interest in learning more about AI or its applications in veterinary medicine (Figure 8, Q14 and Q16, ρ = 0.71), and were moderately correlated with how often they use or interact with AI tools (Figure 8, Q14 and Q11, ρ = 0.68) and how easy they found them to understand and use (Figure 8, Q14 and Q15, ρ = 0.56). A moderate negative correlation was found between how easy it was for students to understand and use AI-driven tools and how often they were overwhelmed by the technology skills required in their studies (Figure 8, Q15 and Q8, ρ = −0.45). The question, “Rate your interest in learning more about AI and its applications in veterinary medicine” strongly correlated with the questions from Section 4, “Do you think AI can help personalize your veterinary learning experience?” (Figure 8, Q16 and Q29, ρ = 0.72) and “How willing are you to experiment with AI tools in your learning process?” (Figure 8, Q16 and Q32, ρ = 0.71).

##### 3.2.1.3 Section 3: standardized clients

**Figure 10:**
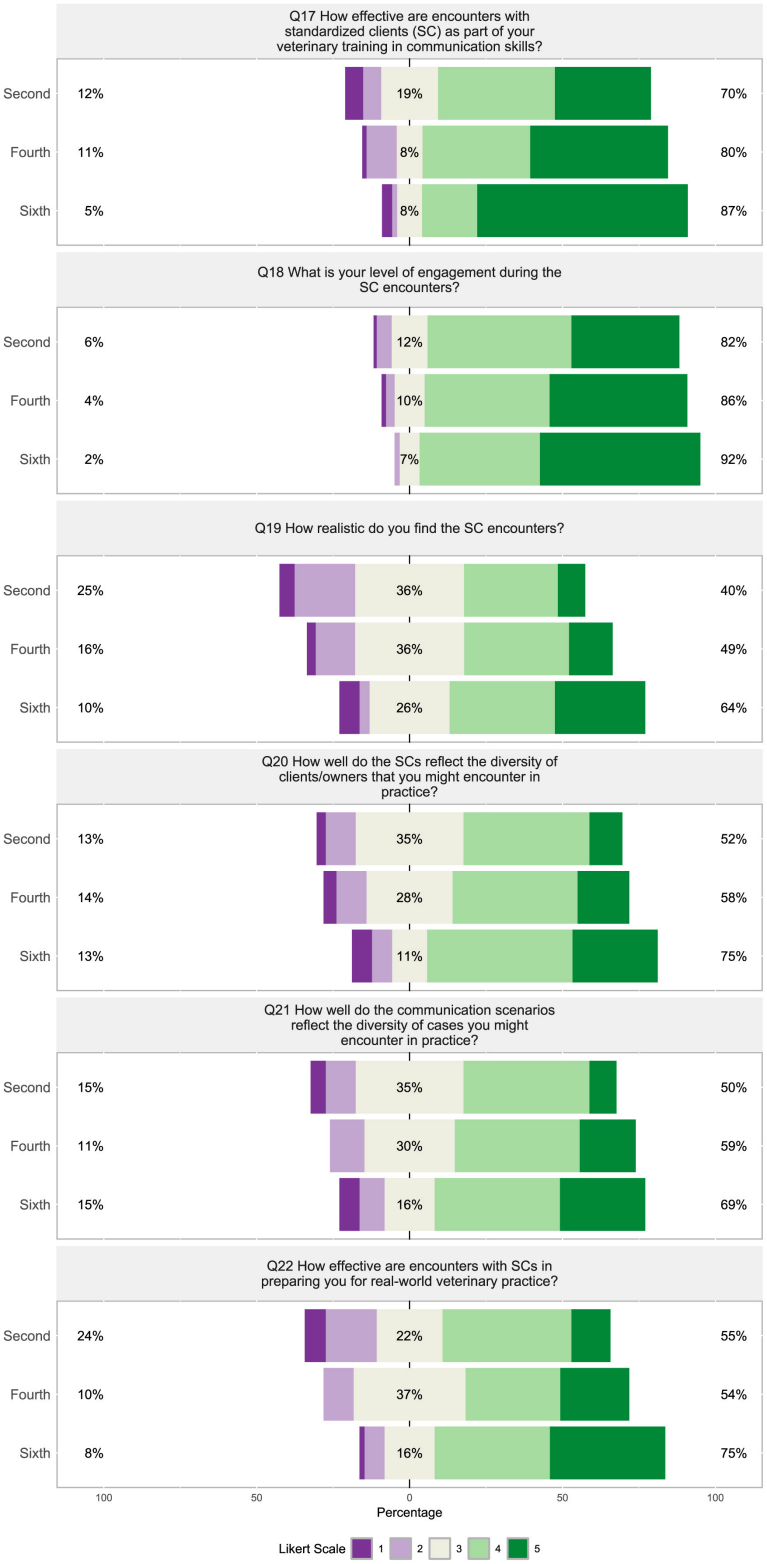
(Q17–Q22): Visualization of the distribution of student responses across different semesters using the Likert scale for responding to questions 17 to 22 in the technology in Section 3: standardized clients survey section. Percentages on the left are more negative responses (Likert scale value 1 and 2: e.g., “not effective” and “not at all realistic”). The middle section shows the percentage of neutral responses (Likert scale value 3). Percentage on the right represent the more positive responses (Likert scale value 4 and 5: e.g., “very effective” and “very realistic”). Table 3 displays the Likert response options.

**Figure 11:**
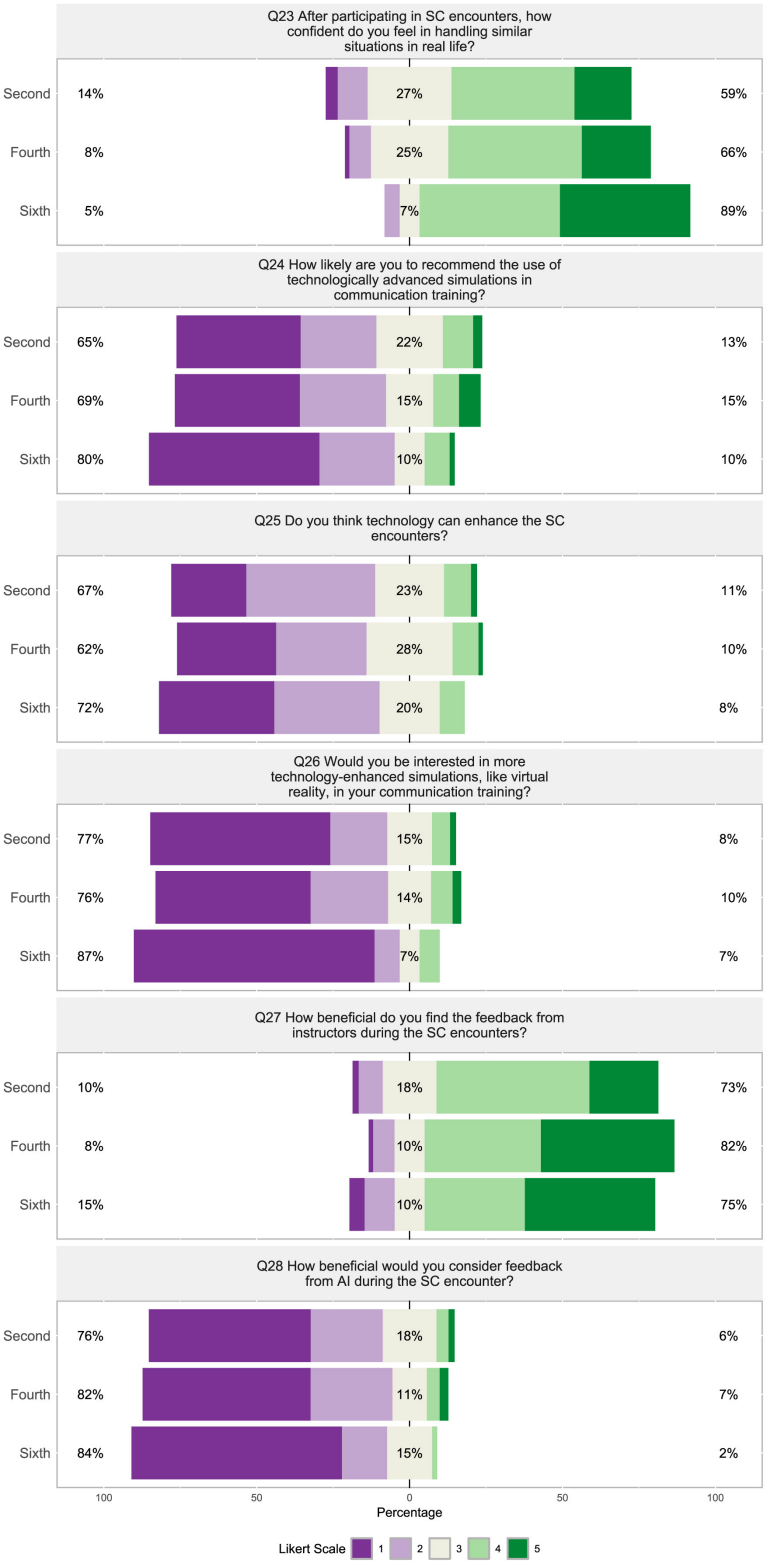
(Q23–Q28): Visualization of the distribution of student responses across different semesters using the Likert scale for responding to questions 23 to 28 in the technology in Section 3: standardized clients survey section. Percentages on the left are more negative responses (Likert scale value 1 and 2: e.g., “not effective” and “not at all realistic”). The middle section shows the percentage of neutral responses (Likert scale value 3). Percentage on the right represent the more positive responses (Likert scale value 4 and 5: e.g., “very effective” and “very realistic”). Table 3 displays the Likert response options.

Students were overall positive toward SC encounters with 70%−87%, believing the encounters were “effective” to “very effective” (Figure 10, Q17), 82%−92% being “quite engaged” to “very engaged” (Figure 10, Q18), 59%-89% being “confident” to “very confident” in handling situations in real life (Figure 11, Q23), and 73%−82% believing feedback from instructors during SC encounters was “beneficial” to “very beneficial” (Figure 11, Q27). Overall students felt SC client encounters were “moderately realistic” (26%−36%) to “quite a bit realistic” or “very realistic” (40%−64%), and that the diversity of TTU SCs reflected the diversity of clients/owners that would be encountered in practice with 52%−75% believing it was “quite a bit” to “very well” (Figure 10, Q19 and Q20). Students also agreed “quite a bit” to “a great deal” (50%−69%) that the communication scenarios were reflective of the breath of cases they would encounter in practice (Figure 10, Q21).

Students were much less positive about technology within the veterinary communications program, with 65%−80% “not at all likely” to “slightly likely” to recommend the use of technologically advanced simulations (Figure 11, Q24), 62%−72% believing that technology can “not at all” or “a little” enhance SC encounters (Figure 11, Q25), 76%−87% being “not at all” to “slightly interested” in more technology-enhanced simulations like virtual reality being incorporated into communication training (Figure 11, Q26), and 76% to 84% believing that feedback from AI during the SC encounter would be “not beneficial” to “only slightly beneficial” (Figure 11, Q28).

A strong correlation was found between how students viewed the realism of SC encounters and how effective the encounters are for preparing them for real-world veterinary practice (Figure 8, Q19 and Q22, ρ = 0.71). Similarly, how students view the diversity of SCs compared to the diversity of clients/owners they may encounter in practice strongly correlated with how well the communication scenarios reflected the diversity of cases they may encounter in practice (Figure 8, Q20 and Q21, ρ = 0.76). The level of students' engagement was weakly negatively correlated with how interested they were in technology-enhanced simulations like virtual reality (Figure 8, Q18 and Q26, ρ =−0.19) and how beneficial they consider feedback from AI during an SC encounter (Figure 8, Q18 and Q28, ρ = −0.19). Questions surrounding the effectiveness (Q17, Q22) and realism (Q19) did not significantly correlate to questions about technology (Q24–Q26) and AI feedback (Q28) within the communications program (Figure 8). Question 18, “What is your level of engagement during the SC encounters?” with SCs did not significantly correlate to the use technology in simulations (Q24) nor if technology can enhance SCs encounters (Q25). Figure 8 shows how the questions in this section were found to inconsistently weakly to moderately correlate with the other sections of the survey (*p* < 0.05).

##### 3.2.1.4 Section 4: expectations and concerns about AI integration in veterinary medical education

**Figure 12:**
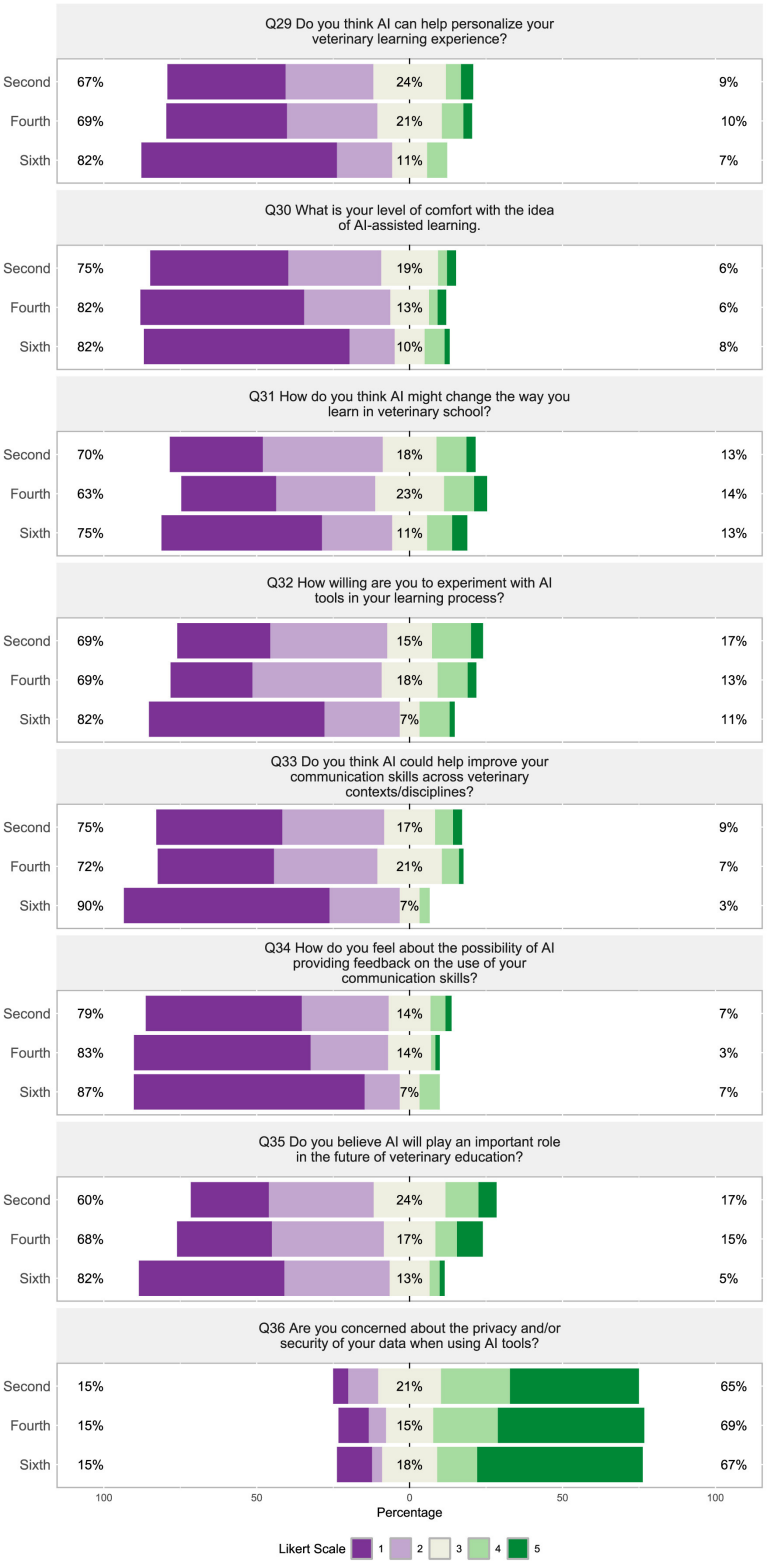
(Q29–Q36): Visualization of the distribution of student responses across different semesters using the Likert scale for responding to questions in the technology in Section 4: expectations and concerns about AI integration in veterinary education and communication survey section. Percentages on the left are more negative responses (Likert scale value 1 and 2: e.g., “not at all willing” and “not at all”). The middle section shows the percentage of neutral responses (Likert scale value 3). Percentage on the right represent the more positive responses (Likert scale value 4 and 5: e.g., “a great extent” and “very significant”). Table 4 displays the Likert response options.

Overall, students appear to have a negative sentiment about AI integration in their veterinary program. Sixty-seven to 82% of students felt AI could “not at all” or only “a small extent” personalize their learning experience (Figure 12, Q29) and 69%−82% were “not at all willing” or “slightly willing” to experiment with AI tools in their learning processes (Figure 12, Q32). Seventy-five to 82% of students were “not comfortable” to only “a little comfortable” with the idea of AI-assisted learning (Figure 12, Q30), while 63%−75% felt AI would “only slightly” or “not at all” change the way they learn in veterinary school (Figure 12, Q31). Students were not open to AI providing feedback on their communication skills, with 79%−87% “not very comfortable” or “slightly comfortable” with this possibility (Figure 12, Q34), while concurrently 72%−90% felt AI could “not at all” or “a little” help improve their communication skills across veterinary contexts/disciplines (Figure 12, Q33). Sixty to 82% believe AI will “not at all” or “only slightly” play an important role in the future of veterinary education (Figure 12, Q34). Nearly two-thirds of students (65% to 69%) were “quite a bit” or “a great deal” concerned about the privacy and security associated with their data when using AI tools.

Nearly all the questions in section four were moderately (ρ = 0.55) to strongly (ρ = 0.77) significantly correlated with each other (Figure 8, Q29 through Q35), except question 36, “Are you concerned about the privacy and/or security of your data when using AI tools?” This question about privacy and/or security was moderately, negatively correlated with “Do you think AI can help personalize your veterinary learning experience?” (Figure 8, Q36 and Q29, ρ = −0.42), “What is your level of comfort with the idea of AI-assisted learning?” (Figure 8, Q36 and Q30, ρ = −0.42), and weakly correlated with the other questions (Figure 8, Q31–Q35). Question 29, “Do you think AI can help personalize your veterinary learning experience?” and question 32, “How willing are you to experiment with AI tools in your learning process?” strongly correlated to question 16, “Rate your interest in learning more about AI and its applications in veterinary medicine?” (Figure 8, Q29 and Q16, ρ = 0.71; Q32 and Q16, ρ = 0.72).

#### 3.2.2 Semester differences

Out of the 36 questions, student Likert responses were statistically different by semester on 16 questions.

##### 3.2.2.1 Section 1: technology and education

**Figure 13:**
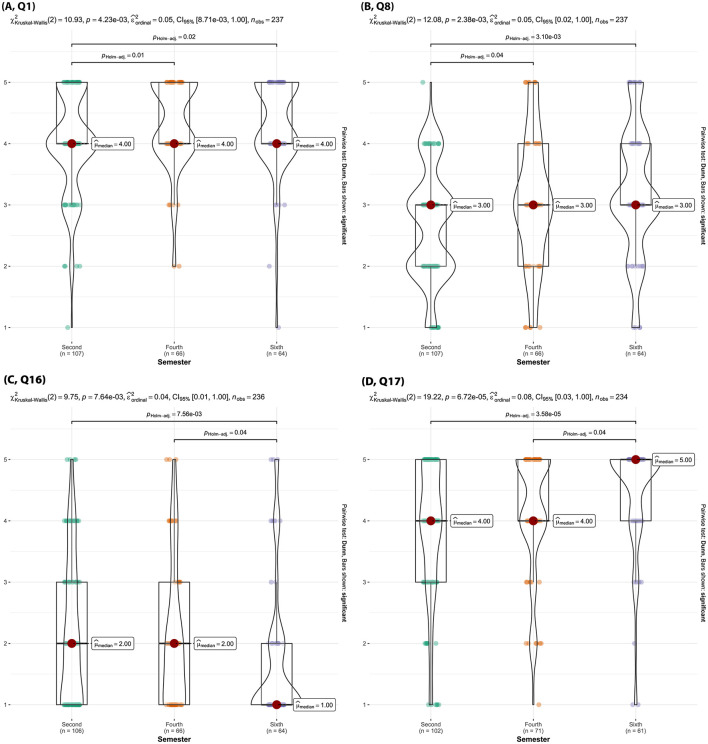
Violin plots of the Kruskal-Wallis test results for [**(A)**, Q1] “How much do you rely on online resources for your veterinary education?”, [**(B)**, Q8] “How often do you feel overwhelmed by the technology skills required in your studies?”, [**(C)**, Q16] “Rate your interest in learning more about AI and its applications in veterinary medicine.” [**(D)**, Q17] “How effective are encounters with standardized clients (SC) as part of your veterinary training in communication skills?” by semester. Significant findings between semesters are denoted by a line with the reported Holms-adjusted *p*-values.

In response to the question “How much do you rely on online resources for your veterinary career,” second semester students showed significantly different viewpoints compared to fourth and sixth semester students (p _Holm − adj_ < 0.05) (Figure 13A, Q1). Additionally, second semester students significantly differed in their response to the question “How often do you feel overwhelmed by the technology skills required for your studies” compared to fourth and sixth semester students (p _Holm − adj_ < 0.01) with more second students reporting they are “rarely” overwhelmed by the required technological skills (Figure 13B, Q8).

##### 3.2.2.2 Section 2: experience with AI and GPTs

Sixth semester students were significantly less familiar with the term “Artificial Intelligence” (AI) compared to students in the second and fourth semester (p _Holm − adj_ < 0.05, Figure 13C, Q16).

##### 3.2.2.3 Section 3: standardized clients

**Figure 14:**
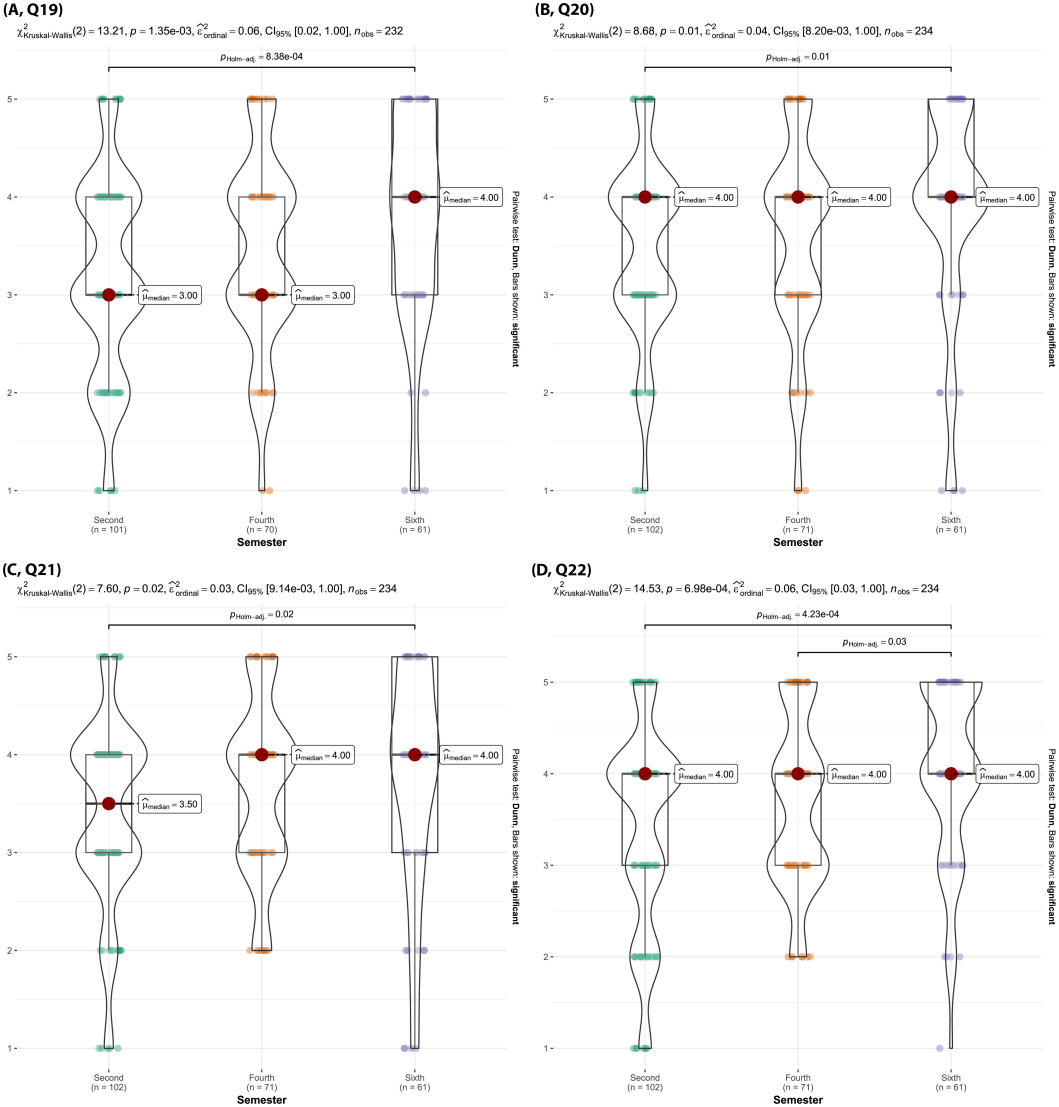
Violin plots of the Kruskal-Wallis test results for [**(A)**, Q19] “How realistic do you find the SC encounters?”, [**(B)**, Q20] “How well do the SCs reflect the diversity of clients/owners that you might encounter in practice?”, [**(C)**, Q21] “How well do the communication scenarios reflect the diversity of cases you might encounter in practice?”, [**(D)**, Q22] “How effective are encounters with SCs in preparing you for real-world veterinary practice.” by semester. Significant findings between semesters are denoted by a line with the reported Holms-adjusted *p*-values.

Sixth semester students rated encounters with SCs as “effective” to “very effective” significantly more often than second semester and fourth semester students did (p _Holm − adj_ < 0.04) (Figure 13D, Q17). Sixth semester students also felt SC encounters were “quite a bit” realistic, whereas second semester felt they were “moderately” realistic (p _Holm − adj_ < 0.01) with fourth semester students perceptions aligning more with the sixth semester (Figure 14A, Q19). Sixth semester students also more strongly believed that both TTU's SCs well reflected the diversity of clients/owners and the diversity of cases they would encounter in practice, compared to second semester students (p _Holm − adj_ = 0.02) (Figures 14B, C Q20, Q21). Moreover, sixth semester students felt SC encounters were significantly more effective at preparing them for real-world veterinary practice compared to second semester and fourth semester students (p _Holm − adj_ < 0.01, Figure 14D, Q22).

**Figure 15:**
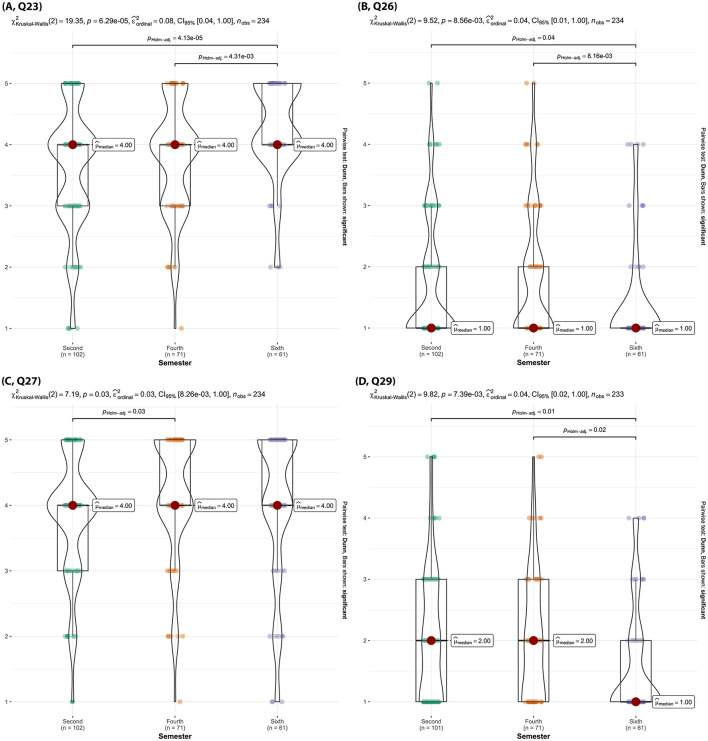
Violin plots of the Kruskal-Wallis test results for [**(A)**, Q23] “After participating in SC encounters, how confident do you feel in handling similar situations in real life?”, [**(B)**, Q26] “Would you be interested in more technology-enhanced simulations, like virtual reality, in your communication training?”, [**(C)**, Q27] “How beneficial do you find the feedback from instructors during the SC encounters?”, [**(D)**, Q29] “Do you think AI can help personalize your veterinary learning experience?” by semester. Significant findings between semesters are denoted by a line with the reported Holms-adjusted *p*-values.

Second semester and fourth semester students felt significantly less confident in their ability to handle situations like those in the SC encounters scenarios compared to sixth semester students (p _Holm − adj_ < 0.01, Figure 15A, Q23). Second semester and fourth semester students showed significantly increased interest in more technology-enhanced simulations like virtual reality compared to sixth semester students (p _Holm − adj_ < 0.04, Figure 15B, Q26). Fourth semester students were significantly more positive about the feedback from instructors during SC encounters being beneficial compared to second semester students (p _Holm − adj_ = 0.03, Figure 15C, Q27).

##### 3.2.2.4 Section 4: expectations and concerns about AI integration in veterinary medical education

**Figure 16:**
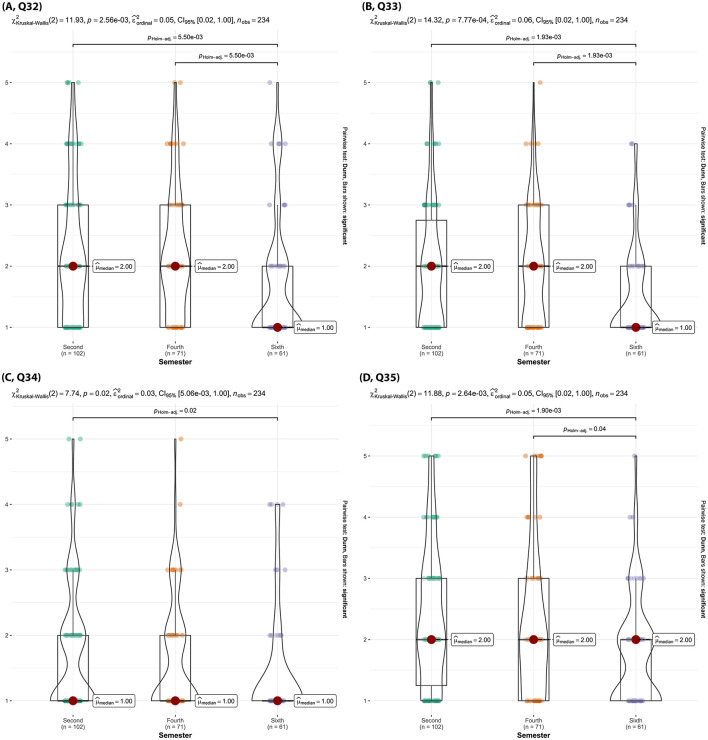
Violin plots of the Kruskal-Wallis test results for [**(A)**, Q32] “How willing are you to experiment with AI tools in your learning process?”, [**(B)**, Q33] “Do you think AI could help improve your communication skills across veterinary contexts/disciplines?”, [**(C)**, Q34] “How do you feel about the possibility of AI providing feedback on the use of your communication skills?”, [**(D)**, Q35] “Do you believe AI will play an important role in the future of veterinary education?” by semester. Significant findings between semesters are denoted by a line with the reported Holms-adjusted *p*-values.

Sixth semester students were significantly more negative about AI's ability to personalize their veterinary learning experience compared to second and fourth semester students (p _Holm − adj_ = 0.01, Figure 15D, Q29) and fourth semester students (p _Holm − adj_ = 0.02, Figure 15D, Q29). Additionally, sixth semester students were significantly less willing to experiment with AI tools in their learning process (Figure 16A, Q32), less open to the idea that AI could help improve their veterinary communication skills (Figure 16B, Q33), and believed significantly less in AI's potential to play an important role in the future of veterinary education (Figure 16D, Q35) compared to second and fourth semester students (p _Holm − adj_ < 0.05). Sixth semester students were also much less comfortable with the possibility of AI providing feedback on their communication skills compared to second semester students (p _Holm − adj_ = 0.02, Figure 16C, Q34).

#### 3.2.3 Career interest differences

**Figure 17:**
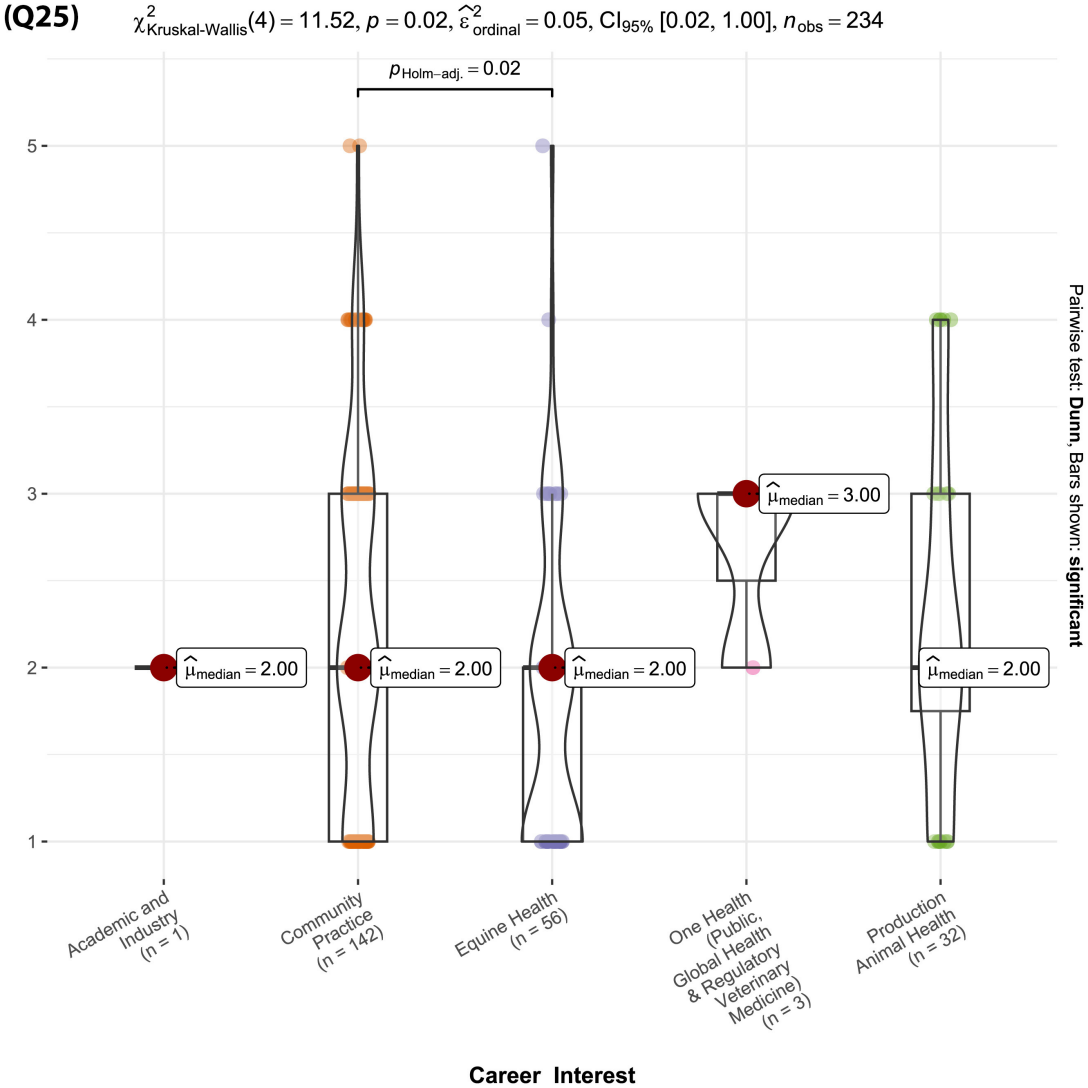
(Q25): Violin plots of the Kruskal-Wallis test results for “Do you think technology can enhance the SC encounters?”—showed a significant difference between students planning to enter community practice and those planning to enter equine practice. Significant findings between students who plan to enter community practice and those planning to enter equine practice are denoted by a line with the reported Holms-adjusted *p*-values.

Out of the 36 survey questions, only one question—“Do you think technology can enhance the SC encounters?”—showed a significant difference between students planning to enter community practice and those planning to enter equine practice (p _Holm − adj_ = 0.02, Figure 17, Q25). The responses of the single student interested in academia and industry were not included in the analysis.

#### 3.2.4 Generational differences

No significant differences were observed in the responses to any question among the Gen Z, Millennial, or Gen X generations.

## 4 Discussion

Our study examined veterinary students' perceptions of AI-technology in veterinary education, particularly regarding the teaching, learning and practice of communication skills. As expected, given current enrollment trends, the majority of participants (58%) were members of Generation Z (born between 1995 and 2012), with 21.5% identifying as Millennials and a very small percentage (0.008%) belonging to Generation X. We recognize that generational differences in beliefs, attitudes, values, motivators and personality traits exist and appreciate that at the heart of generational changes lies the introduction and use of technology (37, 38). While Millennials typically engage with technology mainly for entertainment, Generation X is more information driven. In contrast, Generation Z, often described as “Millennials on steroids” or “iGenZ,” spends significant time daily in front of screens, communicates via emojis and short texts, and rapidly follows, adopts and becomes proficient with multiple technologies (39).

Our findings show no significant differences in veterinary students' perceptions surrounding the use of AI in veterinary medical education and specifically for communication skills training among Gen Z, Millennial or Gen X generations. Despite the heavy use of online resources among veterinary students and about half of our student population reporting that digital tools improved their learning, most expressed little interest in learning more about AI. They also found AI-driven tools challenging to understand and unlikely to be useful for enhancing communication skills. Nearly two-thirds of the veterinary students share concerns surrounding privacy and security of their information/data and these students felt most strongly that AI would not be able to personalize their veterinary learning experience.

### 4.1 Veterinary students' perceptions toward technology in education

There are several motivations for integrating technology into education, including increased accessibility, flexibility, and convenience, as well as improved learning outcomes, motivation, self-control, and interactivity among learners (40, 41). Consistent with studies on medical students' perceptions of technology and e-learning (42) most veterinary students in our study heavily relied on online resources. However, it was surprising to find that over 50% of students rarely explore new technological tools independently beyond what is provided in their courses. This may be related to their reported lack of confidence in learning new technologies and outside of video tutorials on YouTube and other social media platforms (43), there is a general disinterest in using other types of technology during the learning process. Evidence suggests that educators often overestimate students' technical skills, overlooking their limited digital knowledge and abilities in advanced computing functions that may be required for coursework (44). Similarly, veterinary students frequently reported that they are unlikely to recommend the use of digital learning tools to their peers.

### 4.2 Perceived experience with AI in education

The growing utilization of AI in classrooms presents significant opportunities alongside potential challenges and risks in education (4, 45, 46). Modern AI-driven tools, such as Chatbots, are designed to enhance teaching and learning by engaging with users and providing sophisticated, tailored responses. ChatGPT, a generative pre-trained transformer model, leverages natural language processing (NLP) to interpret, engage, and generate human-like-like responses in real-time conversations. It can add new content, offer suggestions, respond to follow-up questions, identify errors, and even understand social and emotional cues and queries (47). For students, ChatGPT can provide feedback, and assist with writing assignments, while it supports teachers by generating course content, syllabi, presentations, quizzes, and more (45). Additionally, in veterinary clinical practice, LLMs can compile data from unstructured veterinary records to help enhance patient outcomes and provide data in a format usable for animal disease surveillance (12). Our study findings demonstrate that veterinary students are generally aware of AI technology in everyday applications like social media and predictive text. However, while they are familiar with the term AI, they seldom use, interact with, or show interest in learning more about AI and its applications in veterinary education. These findings suggest that students may lack the technical skills to independently explore the use of technology and concurrently that they may be disconnected from recognizing the importance of understanding the use of AI in veterinary education and veterinary practice. Ethical considerations surrounding the use of such technologies in education and veterinary medicine necessitate clear guidelines to ensure acceptable and ethical usage, which are currently lacking in many institutions, potentially hindering the full use of AI (12, 47).

### 4.3 Standardized clients for practicing veterinary communication skills

Small-group training that incorporates feedback and SCs is an effective approach in improving communication skills (22, 48). Likewise, our study findings reported that SC encounters are effective (69%−86%) and engaging (82%−91%) in practicing communication skills resulting in enhanced confidence when handling similar encounters in practice (58%−86%). Furthermore, veterinary students found the SC encounters quite realistic (40%−64%) aligning with evidence supporting authenticity during client encounters (49, 50). Surprising, our findings reported that SC encounters reflected the diversity that they may encounter in practice, yet evidence strongly delineates the need for expanding diversity across communication programs (51). Surveying AVMA-COE accredited institutions showed that the majority of SCs were primarily English speaking (77%), white (90.4%), non-Hispanic/Latinx (98.6%), female (57%), and over age 56 (64%) (51). SC demographics at TTU SVM are much similar with 84% reporting white, female (78%), heterosexual (100%), over age 30 and 56 (10% and 89% respectively), and retired (98%). Concurrently, our communication program explores cultural competence and exposes students to a diverse context as well as a variety of cases across species which may be contributing to a perceived adequate SC representation.

### 4.4 Perceived advantages of introducing emerging technology in communication training

Our study reported that most veterinary students (66%−81%) found that technology, particularly technologically advanced simulation, cannot fully replace experiential practice with SCs. This finding aligns with results from studies that integrated technology using a blended approach (52, 53). There is mixed evidence about the use of emerging technologies. Some studies evaluated Virtual Patients (VP) and found their experience to be as effective as practicing with SCs, with VPs offering added visual and behavioral advantages (54, 55). However, other reports highlighted limitations when working with VPs. Unlike other studies, the majority (76%−88%) of veterinary students in our research showed little interest in technology-enhanced simulations, such as VPs, and emphasized the importance of expert feedback (73%−83%), while expressing little confidence in the benefits of AI-generated feedback (76%−84%). Other studies noted that both instructors and students appreciated AI feedback but acknowledged the importance of prioritizing instructor feedback generated before AI feedback. Additionally, students recognized the limitations of AI in interpreting non-verbal communication, attitudes and beliefs which can be complex (30).

### 4.5 Difference in perception across academic years

Study results indicated that sixth-semester students reported a greater appreciation for and understanding of experiential learning with SCs when practicing communication skills. They recognized the effectiveness, realism, and diversity in these interactions, which reflected the challenges that they would face in clinical practice. This exposure gave them the confidence to handle similar situations, more so than students in earlier semesters, particularly those in semester two. Research evidence supports that senior students acknowledged effective communication as a core clinical competency for a successful veterinary career, highlighting the importance of communication skills training during both pre-clinical and clinical curricula (56, 57). In contrast, previous studies have shown that 1^st^-year-students often report inflated levels of communication competence (58), with perhaps less appreciation for the necessity of communication skills training which may also explain our second semester student views.

Interestingly, despite growing evidence of veterinary students' acceptance of AI in veterinary medicine (59), sixth-semester students expressed a strong lack of interest and reluctance toward using emerging technologies for communication training, particularly regarding the use of AI. Studies involving medical students and professionals have reported similar concerns, with fears that AI could replace physicians and lead to new professional liabilities. This personal and professional apprehension has contributed to a resistance to exploring AI's potential in academic and clinical settings (60). Our sixth-semester students informally shared that they were opposed to AI based on fears that AI would potentially replace the use of SCs in teaching and learning communication skills.

The fear of AI replacing SCs, may have greatly contributed to the strong lack of interest and reluctance in incorporating AI into their veterinary education and veterinary communication training. Our sixth semester students began their veterinary education prior to the advent of readily accessible LLMs like ChatGPT-3.5 released in November 2022, and only recently have specialized LLM veterinary tutors begun to be developed. This suggests perhaps the fears or replacement of SCs are based on the lack of exposure to AI as an educational tool. This is supported by second semester students being more interested in technology-enhanced simulations and having AI tutor tools such as VetClinPathGPT, https://chatgpt.com/g/g-rfB5cBZ6X-vetclinpathgpt, available for utilization while studying for their courses.

### 4.6 Limitations

The findings of this study reflect the perspectives of veterinary students at Texas Tech University School of Veterinary Medicine and are not fully generalizable to other veterinary institutions. The lack of prior experience with AI integration in the curriculum may have heightened skepticism among students, compounded by unclear guidelines surrounding AI use, concerns about the authenticity of their work, and perceived risks associated with this technology. While the study effectively captured concerns about data privacy, it was not designed to investigate or propose solutions to these issues. A broader, multi-institutional approach that compares student responses across academic years and institutions could provide a more comprehensive understanding of perceptions related to AI in veterinary communication, particularly in communication skills training. Future qualitative studies could further explore deeper into the underlying reasons for negative attitudes and better understand institutional strategies to address data privacy concerns, reduce apprehensions, and support the thoughtful adoption of AI to support veterinary education training.

## 5 Conclusions

This study integrated artificial intelligence (AI) into the training of veterinary communication skills, offering valuable insights into the applicability and perception of AI within veterinary education. Our findings reveal that while students recognize the prevalence of AI in everyday technology, their familiarity and comfort with AI-driven tools in educational settings, particularly in communication training, remains limited. Furthermore, the data suggest that upper-semester students are less open to adopting AI-based tools in communication training compared to those in earlier semesters, likely due to their greater reliance on experiential learning with standardized clients (SCs). Future studies should focus on multi-institutional studies to assess the generalizability of these findings. Additionally, qualitative data and a longitudinal study (repeated measures with continued use of AI generated cases) would be additional opportunities for understanding how this technology and methodology could be used in a curriculum. Encouraging students to engage with AI in a structured, supportive environment may help alleviate some of the apprehension seen, paving the way for AI to play a more prominent role in veterinary education.

## Data Availability

The dataset and the R code used for statistical analysis for this study can be found in the AI_VeterinaryCommunications Git Hub Repository available at https://github.com/MicroBatVet/AI_VeterinaryCommunications (61).
